# Longitudinal metabolomic profiling of biogenic amines in plasma and CSF, and their correlation, reveals sex-specific and age changes in TgF344 Alzheimer’s disease transgenic and wildtype rats

**DOI:** 10.1186/s12987-026-00811-8

**Published:** 2026-05-09

**Authors:** Chunyuan Yin, Imke Nelen, Amy Harms, Robin Hartman, Sabine Bos, Charlotte Nijgh-van Kooij, Thomas Hankemeier, Alida Kindt, Elizabeth de Lange

**Affiliations:** 1 Division of Systems Pharmacology and Pharmacy, Leiden Academic Centre for Drug Research, Leiden University, Leiden, The Netherlands; 2 Metabolomics and Analytics Centre, Leiden Academic Centre for Drug Research, Leiden University, Leiden, The Netherlands

**Keywords:** Alzheimer’s disease, Metabolomics, Amines, TgF344-AD transgenic rat model, CSF, Plasma

## Abstract

**Background:**

Alterations in amine metabolism have been implicated in Alzheimer’s disease (AD), but the relationships between plasma and cerebrospinal fluid (CSF) amine levels remain insufficiently understood.

**Aim:**

To investigate longitudinal changes in amines in plasma and CSF, as well as their cross-matrix correlations, in male and female TgF344-AD transgenic rats compared with wild-type (WT) controls.

**Method:**

LC-MS-based targeted metabolomics was used to quantify 60 plasma amines and 55 CSF amines in male and female TgF344-AD and WT rats at 12, 25, 50 and 85 weeks of age. Generalized linear models, Pearson correlations, and Fisher’s r-to-z transformation were applied for statistical analysis.

**Results:**

In plasma, age- and sex-associated differences were observed. At 25 weeks, three amines (4-hydroxy-proline, homocitrulline, and hydroxylysine) showed significantly increased levels in male TgF344-AD rats after multiple-testing correction. Additional trend-level changes were observed at 12, 50, and 85 weeks. In CSF, no amines passed the significance threshold after multiple-testing correction, although descriptive age- and sex-associated patterns were observed, with earlier changes in males and later-stage trends in females. CSF–plasma correlations tended to be stronger in TgF344-AD rats than in WT rats, with relatively strong correlations for alpha-aminobutyric acid, citrulline, N6,N6,N6-trimethyl-lysine, and putrescine.

**Conclusions:**

Body fluid, age- and sex-dependent amine alterations in CSF and plasma of TgF344-AD rats compared to WT controls provide important insights into AD disease processes and may aid early diagnosis and therapeutic targeting.

**Supplementary Information:**

The online version contains supplementary material available at 10.1186/s12987-026-00811-8.

## Introduction

Alzheimer’s disease (AD) is the leading cause of dementia worldwide, characterized by progressive memory loss and cognitive decline [1]. Its hallmark pathological features include the accumulation of amyloid-beta plaques in the extracellular space and the formation of neurofibrillary tangles composed of hyperphosphorylated tau within neurons [2, 3]. These processes result in neuronal damage, synaptic dysfunction, and brain atrophy, which begin years to decades before clinical symptoms emerge [4]. While recent therapeutic advancements, such as the FDA-approved Aduhelm (aducanumab) and Leqembi (lecanemab), target amyloid pathology, their effectiveness is largely restricted to early disease stages [5, 6]. This underscores the urgent need for early biomarkers and a deeper understanding of AD-related metabolic alterations to improve diagnosis and therapeutic strategies, particularly for later disease stages where current treatments offer limited benefits.

Biogenic amines play important roles in various physiological processes, including acting as neurotransmitters, e.g., dopamine, glutamate, or serotonin [7], regulating blood pressure, e.g., histamine or tyramine, and contributing to cellular signaling and metabolic pathways, all of which are vital for maintaining brain function and systemic homeostasis [8, 9]. Alterations in amine metabolism have been increasingly implicated in neurodegenerative diseases, particularly AD [10, 11]. For instance, the amino acid glutamate is a key excitatory neurotransmitter and essential for synaptic transmission, but its dysregulation can lead to excitotoxicity, a process causing neuronal death, which is characteristic of AD progression [12, 13]. Gamma-aminobutyric acid (GABA), an inhibitory neurotransmitter, is also disrupted in AD, contributing to cognitive deficits and neuronal dysfunction [14]. In addition to neurotransmission, branched-chain amino acids (BCAAs) are involved in maintaining brain homeostasis, and disturbances in their metabolism may exacerbate neurodegeneration [15]. Evidence from both human and animal studies suggests that altered levels of amines such as serine, glycine, and alanine in cerebrospinal fluid (CSF) and plasma are associated with both early and late stages of AD, making them promising candidates for biomarkers of early disease development [16–18].

Direct brain sampling is only feasible postmortem, while CSF is the body-fluid in closest contact with the brain which allows it to best reflect brain-specific metabolic and pathological changes. However, its invasive collection limits widespread clinical use [19]. Plasma, although being more accessible and cost-effective in sampling, mainly reflects systemic metabolism and peripheral processes, but may partly reflect central processes. Therefore, plasma metabolomic profiling could serve as a valuable tool for tracking both systemic and brain-related metabolic disturbances during AD progression. Recent metabolomics studies have shown that certain metabolic changes in plasma closely mirror those in CSF, including disturbances in amino acid and polyamine metabolism, lysine degradation, and energy pathways [16, 20]. These findings highlight the potential of plasma profiling to reflect central metabolic alterations, supporting its role as a complementary biofluid for biomarker discovery. However, so far, only few studies have addressed changes in amine profiles in the CSF and plasma in AD [16, 21], and more information is needed to understand the role of amines in AD progression. Longitudinal studies comparing individuals with AD to control groups should be conducted, since AD progression is closely associated with aging. These studies should also examine potential sex and gender differences [22].

Direct plasma-CSF correlations could support the use of plasma amines as potential non-invasive biomarkers for AD-related metabolic alterations. In our previous work we characterized age-, sex-, and AD-specific lipidomic alterations in plasma from TgF344-AD rats [22]. In this study, we explored CSF and plasma amine alterations of the same male and female TgF344-AD rats and their wild-type (WT) littermates at 12, 25, 50 and 85 weeks. Using targeted UPLC-MS we quantified amines in plasma and CSF, and compared their levels in the different age groups, sex and across biofluids in WT vs. transgenic AD rats.

## Materials and methods

### Animals and sample collection

TgF344-AD rats and their age-matched wild-type (WT) Fischer F344 littermates were obtained and bred as previously described [22]. Briefly, this longitudinal study included 75 TgF344-AD rats and 74 WT littermates of both sexes, sampled at 12, 25, 50, and 85 weeks (detailed in Table 1). Housing conditions, including 12 h/12 h light-dark cycles, controlled temperature (21 ± 2 °C), humidity (40–60%) and standard rodent diet, were maintained as previously reported. Animal protocols were approved by the Leiden University Animal Welfare Body (AWB: AVD1060020171766). Blood and CSF samples were collected following established procedures [22]. Blood was drawn from the left ventricle of the heart into ethylenediaminetetraacetic acid (EDTA) tubes, and CSF was aspirated directly from the Cisterna magna. Both sample types were centrifuged at 2300G for 10 min at 4 °C, aliquoted, and stored at -80 °C until further analysis.

**Table 1:** Number of CSF and plasma samples for males and females at different ages in the WT and TgF344-AD rat groups; w = weeks

Age		WT rats				TgF344-AD rats			
		12 w	25 w	50 w	85 w	12 w	25 w	50 w	85 w
CSF	female	5	7	8	9	6	10	10	11
	male	10	11	8	9	7	9	8	7
Plasma	female	7	8	8	11	8	10	10	12
	male	9	12	9	10	9	9	9	8

### Amine measurement

Plasma and CSF samples were analyzed at the Biomedical Metabolomics Facility Leiden (BMFL), using an assay which quantifies biogenic amines using an AccQ-Tag derivatization method, adapted from the Waters protocol as previously described [23].

For each analysis, samples containing 5.0 µL of plasma or 10 µL of CSF were mixed with 5.0 µL of a spiked internal standard solution. Proteins were then precipitated by the addition of methanol, after which the samples were dried in a SpeedVac (Thermo). Residues were reconstituted in a borate buffer (pH 8.8) with AQC reagent. After the reaction, the samples were acidified with 10 µL of 20% formic acid and transferred to autosampler vials, then placed in an autosampler tray and cooled to 4 °C until injection. Finally, 1.0 µL of the reaction mixture was injected into the UPLC-MS/MS system for targeted quantification of amines. Chromatographic separation was performed using an Agilent 1290 Infinity II LC System equipped with an AccQ-Tag Ultra column (Waters), operating at a flow rate of 0.7 mL/min over an 11-minute gradient. The UPLC was coupled to a triple quadrupole mass spectrometer (AB SCIEX Qtrap 6500) utilizing electrospray ionization. Analytes were detected in the positive ion mode and monitored using Multiple Reaction Monitoring (MRM) with nominal mass resolution. The acquired data were evaluated using MultiQuant Software for Quantitative Analysis (AB SCIEX, Version 3.0.2), by integration of assigned MRM peaks and normalization using the selected internal standards. For amino acid analysis, 13C15N-labeled analogs were utilized. For other amines, the closest-eluting internal standard was employed. Blank samples were used to assess background signal. Batch correction was performed using median normalization of the pooled quality control samples [24]. A total of 55 amines features from CSF and 60 from plasma with relative standard deviation of the quality control samples < 30% passed the quality evaluation and were included in the statistical analysis.

### Statistical analysis

All statistical tests were performed in R Studio (Version 2024.9.0.375). Amines passing quality evaluation were log2 transformed before analysis to normalize data distribution and reduce heterogeneity of variance.

#### Generalized linear regression model

Univariate differences of amines in CSF and plasma between TgF344-AD and age-matched WT rats were assessed using generalized linear regression models (Gaussian family). For each metabolite, the log2-transformed metabolite concentration was used as the dependent variable. Genotype (TgF344-AD vs. WT), sex, and age (12, 25, 50, and 85 weeks) were included as explanatory variables. Analyses were conducted for both overall comparisons and subgroup analyses. Metabolite estimate values (β coefficients) and corresponding p-values were extracted from the model summaries. P-values were adjusted for multiple comparisons using the Benjamini-Hochberg method to control the false discovery rate (FDR). Results with nominal *p* < 0.05 were considered trend-level findings, whereas metabolites with FDR-adjusted q < 0.05 were considered statistically significant. Data visualization was performed using the `ggplot2` package in R.

#### Correlation analysis

To investigate the relationships between CSF and plasma metabolites, Pearson correlation coefficients were calculated using “cor.test” function for each metabolite across different disease groups (WT and TgF344-AD). Correlation analyses were classed by age (12, 25, 50, and 85 weeks) and sex (male and female) to identify age- and sex-specific patterns.

Correlations with p-values (*p* < 0.05) were considered trends while those with FDR-adjusted q-values (q < 0.05) were labelled as significant. Correlation strength was interpreted based on the magnitude of the correlation coefficient, with strong correlations defined as |R| ≥ 0.6 and moderate correlations as 0.4 ≤ |R| < 0.6.

Correlation heatmaps were generated using the `pheatmap` package in R, with hierarchical clustering applied to highlight metabolite groups exhibiting similar correlation patterns. Correlations with |R| < 0.35 were considered noise and not appropriate for visualization and were therefore left blank in the heatmap. Grouping in the heatmap is by both sex (male and female) and age (12, 25, 50, and 85 weeks), allowing for the exploration of sex- and age-dependent correlation patterns between CSF and plasma metabolites. Comparisons were performed separately for WT and TgF344-AD rats to assess disease-specific correlation differences across sex and age.

Differential correlation analysis was performed using Fisher’s r-to-z transformation to generate z-scores and associated p-values for the difference between AD and WT correlation coefficients for each metabolite in general and separated by gender and age.

## Results

### Age- and sex-specific alterations in CSF and plasma amines profiles in TgF344-AD and wildtype rats

#### Alterations in CSF biogenic amine profiles

**Fig. 1:**
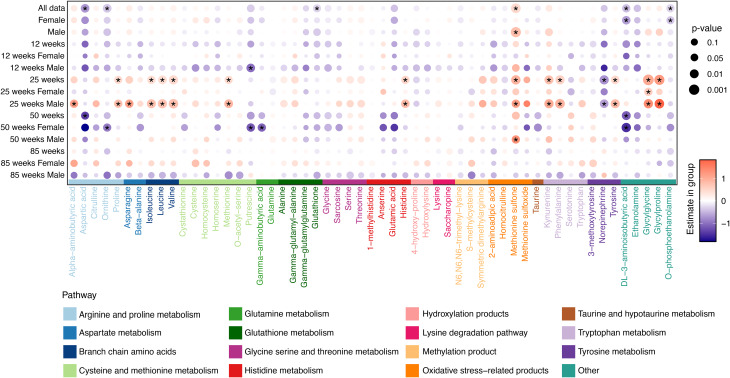
Estimated differences in CSF amines comparing TgF344-AD with age-matched wildtype (WT) rats based on generalized linear regression models (GLMs). Each dot represents the estimated group difference (β coefficient), with color indicating the direction of change (orange = higher in TgF344-AD; purple = lower) and dot size reflecting the significance level. Metabolites with nominal significance (*p* < 0.05) are marked with an asterisk (*). No metabolites reached the FDR-adjusted threshold of q < 0.05 in the CSF analysis. Biogenic amines are color-coded according to their associated metabolic pathways, as indicated in the legend. Corresponding p-values and FDR-adjusted q-values for all tested comparisons are provided in Supplementary Table 3

CSF amine differences between TgF344-AD rats and age-matched WT rats, assessed by generalized linear regression models, are summarized in Fig. 1. The figure illustrates estimate group effects (β coefficients) across sex and age, where orange indicates higher levels in TgF344-AD rats and purple indicates lower levels compared to WT rats. Findings reaching an FDR-adjusted threshold of q < 0.05 are considered statistically significant, whereas results with nominal significance (p < 0.05) are interpreted as trends. In the CSF analysis, no metabolites passed the threshold of q < 0.05 after FDR correction.

Overall, the analysis suggested descriptive age- and sex-associated differences in CSF biogenic amines across disease progression.

At *12 weeks*, changes were minimal, with only putrescine showing a decrease in male TgF344-AD rats compared WT rats, while no metabolite changes were observed in female rats.

By *25 weeks*, male TgF344-AD rats showed higher levels of multiple biogenic amine (e.g., alpha-aminobutyric acid, asparagine, glycylglycine, glycylproline, histidine, isoleucine, kynurenine, leucine, methionine, methionine sulfone, phenylalanine, proline, tyrosine, and valine). A decrease was observed for norepinephrine. No metabolite changes were observed in female rats.

In contrast, at *50 weeks*, female TgF344-AD rats displayed decreases in several amines including DL-3-aminoisobutyric acid, gamma-aminobutyric acid, ornithine, and putrescine, while 50-week male TgF344-AD rats showed a trend towards increased methionine sulfone level compared to 50-week male WT rats.

At *85 weeks*, no CSF amines passed *p* < 0.05 between TgF344-AD and WT rats.

To formally evaluate sex-dependent genotype effects across age, generalized linear models including genotype × sex × age interaction terms were fitted. Nominally significant three-way interaction terms (*p* < 0.05) were identified for s-methylcysteine, alpha-aminobutyric acid, and asparagine. However, none remained significant after FDR correction, and no other genotype × sex × age interaction terms were significant. Full interaction results are provided in Supplementary Table 1.

Together, these results describe age- and sex-associated changes in CSF amine levels, with male TgF344-AD rats showing changes at an earlier time point (25 weeks), and females showing later-stage trends (50 weeks) during AD progression.

#### Alterations in plasma biogenic amine profiles

**Fig. 2:**
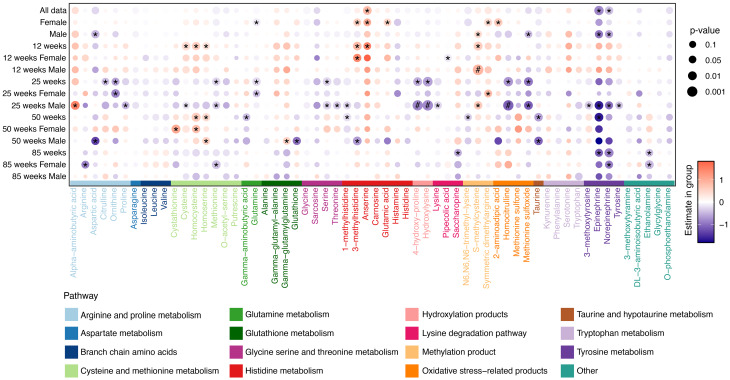
Estimated differences in plasma amines comparing TgF344-AD with age-matched wildtype (WT) rats based on generalized linear regression models (GLMs). Each dot represents the estimated group difference (β coefficient), with color indicating the direction of change (orange = higher in TgF344-AD; purple = lower) and dot size reflecting the significance level. Metabolites with nominal significance (*p* < 0.05) are marked with an asterisk (*), whereas those reaching q < 0.05 are marked with a hash (#). Biogenic amines are color-coded according to their associated metabolic pathways, as indicated in the legend. Corresponding p-values and FDR-adjusted q-values for all tested comparisons are provided in Supplementary Table 4

Plasma amine differences between TgF344-AD rats and age-matched WT rats are summarized in Fig. 2. Findings reaching an FDR-adjusted threshold of q < 0.05 are considered statistically significant, whereas results with nominal significance (p < 0.05) are interpreted as trends. Age- and sex-dependent metabolic differences were observed in plasma.

At *12 weeks*, TgF344-AD rats showed increased levels of 3-methylhistidine, anserine, cysteine, homocysteine, homoserine, and s-methylcysteine compared to WT rats. When stratified by sex, male TgF344-AD rats displayed increases in s-methylcysteine, whereas female rats showed no significant alterations.

At *25 weeks*, three amines (4-hydroxy-proline, homocitrulline, and hydroxylysine) passed the FDR threshold in male TgF344-AD rats, representing the most robust findings observed in plasma at this age. In addition, several other amines showed trends toward higher levels, including 1-methylhistidine, 3-methoxytyrosine, citrulline, cysteine, epinephrine, homoserine, lysine, methionine, methionine sulfoxide, norepinephrine, proline, serine, threonine, and tyrosine, whereas alpha-aminobutyric acid and s-methylcysteine showed lower levels in TgF344-AD rats. These findings were consistent with those observed in the 25-week combined-gender group, suggesting that patterns at 25 weeks were primarily driven by males. In contrast, no metabolites reached statistical significance in females at this age.

At *50 weeks*, male TgF344-AD rats showed trends toward decreased levels of 3-methylhistidine, aspartic acid, glutathione, and taurine, while gamma-glutamylglutamine and homoserine showed trends toward increased levels in. No significant or trend-level changes were detected in females.

At *85 weeks*, female TgF344-AD rats showed trends toward decreased levels in several amines including arginine, ethanolamine, methionine, and norepinephrine, whereas no metabolites met the statistical threshold in males.

Applying the genotype × sex × age interaction model to plasma metabolites identified a nominal three-way interaction for pipecolic acid, which did not pass the FDR threshold, no significant interaction effects were observed for the remaining metabolites (Supplementary Table 2).

Altogether, these results describe age- and sex-associated patterns in plasma amine alterations, with early increases in young TgF344-AD rats and progressive declines at later stages, particular in males. These patterns are consistent with those observed in CSF and indicate systemic manifestations of amine dysregulation during AD progression.

### Pearson correlation between CSF and plasma biogenic amines in TgF344-AD and WT rats

**Fig. 3:**
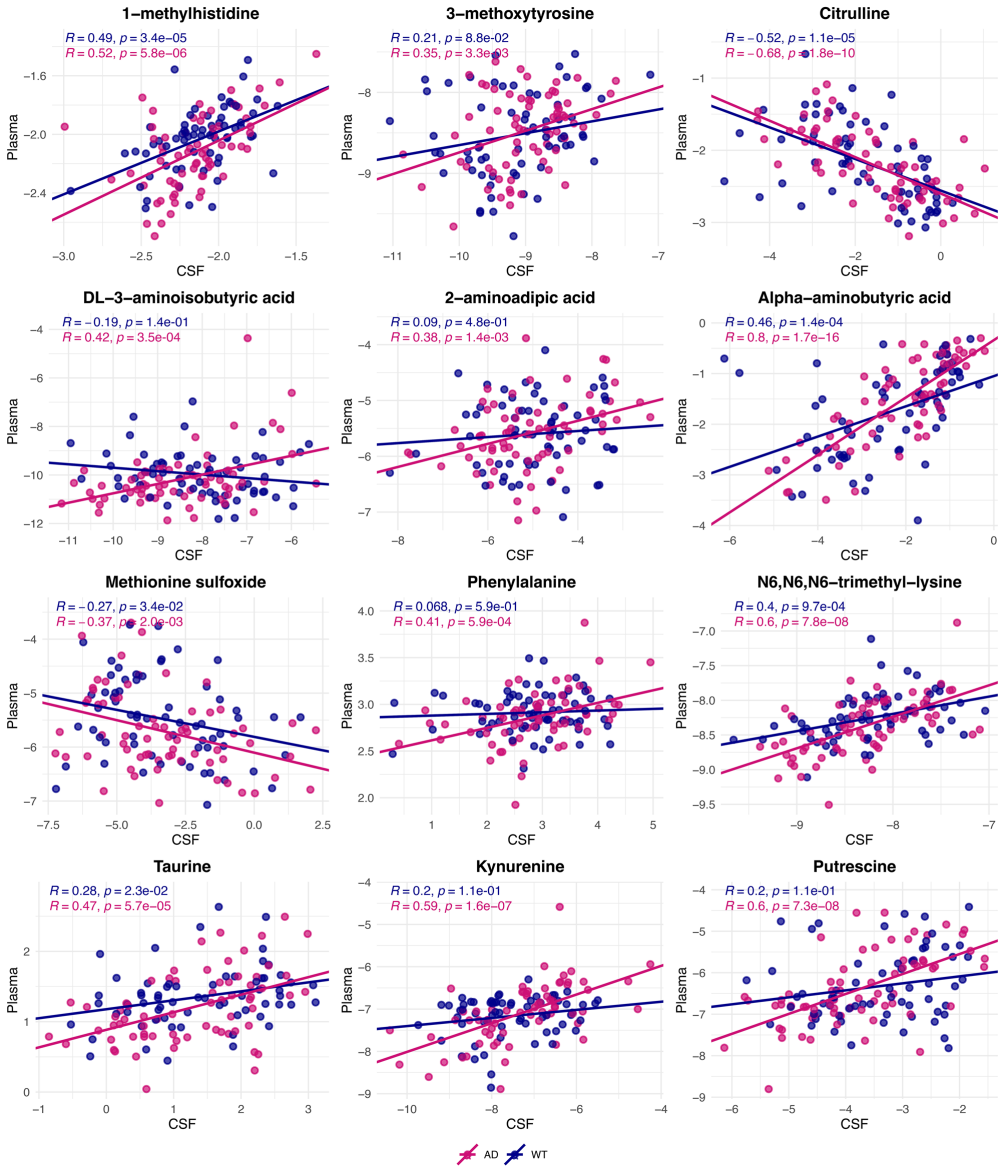
Pearson correlation analysis of CSF and plasma metabolites highlighting correlations within TgF344-AD (dark pink) and WT rats (purple). For each amine, points represent the log2-transformed concentration measured in CSF (x-axis) and plasma (y-axis) for individual rats. Solid lines indicate linear regression fits, and the corresponding Pearson correlation coefficients (R) indicate the strength and direction of the associations between CSF and plasma levels. In TgF344-AD rats, strong correlations were observed for metabolites such as alpha-aminobutyric acid, citrulline, N6,N6,N6-trimethyl-lysine, and putrescine while the WT rats showed moderate correlations for metabolites like 1-methylhistidine, alpha-aminobutyric acid, citrulline, and N6,N6,N6-trimethyl-lysine. Strong correlations are defined as |R| ≥ 0.6, and moderate correlations as 0.4 ≤ |R| < 0.6

CSF-plasma amine correlations across TgF344-AD and WT rats are summarized **in** Fig. 3, which highlights the overall strength and direction of associations between the two biofluids. Of the 53 amines detected in both biofluids, 12 exhibited statistically significant (*q* < 0.05) CSF-plasma correlations in either TgF344-AD or WT rats. The *p*-values and FDR-adjusted *q*-values for these correlations, together with the results of the Fisher’s r-to-z transformation comparing AD and WT groups, are provided in Table 2.

Overall, TgF344-AD rats tended to show stronger Pearson correlations between CSF and plasma amine levels compared to WT rats. Alpha-aminobutyric acid, citrulline, N6,N6,N6-trimethyl-lysine, and putrescine showed relatively strong correlations (|R| ≥ 0.6) in the TgF344-AD rats, while these correlations were only moderate (0.4 ≤ |R|< 0.6) in WT rats. When stratified by sex (Supplementary Tables 6–7), female rats exhibited more pronounced differences in CSF-plasma correlations between TgF344-AD and WT groups, particularly for alpha-aminobutyric acid, citrulline, and N6,N6,N6-trimethyl-lysine. Across ages (Supplementary Tables 8–11), correlation patterns in citrulline evolved dynamically: WT rats showed stronger negative correlation at 25 weeks, while TgF344-AD became clearly more negative at 50 weeks and 85 weeks.

Taken together, these data suggest that some amines, e.g., alpha-aminobutyric acid, N6,N6,N6-trimethyl-lysine, may exhibit stronger CSF-plasma correlations in TgF344-AD rats compared to WT, with some differences observed between sexes. In contrast, other amines, e.g., citrulline, show variation in correlation patterns across age and sex, with differences observed between WT (25 weeks) and TgF344-AD rats (50–85 weeks).

**Table 2:** Top CSF-plasma correlated amines in TgF344-AD and WT rats

Amines	TgF344-AD rats			WT rats			Fisher’s z-value AD/WT		
	*R*_AD_	*p*-value	q-value	*R*_WT_	*p*-value	q-value	z-value	*p*-value	q-value
alpha-aminobutyric acid	0.80	1.71E-16	9.07E-15	0.46	1.38E-04	2.43E-03	4.91	9.10E-07	1.09E-05
N6,N6,N6-trimethyl-lysine	0.60	7.85E-08	1.04E-06	0.40	9.65E-04	1.28E-02	2.1	3.61E-02	8.66E-02
putrescine	0.60	7.30E-08	1.04E-06	0.20	1.13E-01	3.20E-01	3.91	9.20E-05	5.52E-04
kynurenine	0.59	1.58E-07	1.67E-06	0.20	1.15E-01	3.20E-01	3.76	1.70E-04	6.80E-04
1-methylhistidine	0.52	5.76E-06	5.09E-05	0.49	3.36E-05	8.91E-04	0.27	7.86E-01	7.86E-01
taurine	0.47	5.69E-05	4.31E-04	0.28	2.26E-02	1.50E-01	1.72	8.46E-02	1.27E-01
DL-3-aminoisobutyric acid	0.42	3.49E-04	2.31E-03	-0.19	1.40E-01	3.70E-01	5.12	3.05E-07	7.32E-06
phenylalanine	0.41	5.94E-04	3.50E-03	0.068	5.92E-01	8.48E-01	2.91	3.60E-03	1.08E-02
2-aminoadipic acid	0.38	1.44E-03	7.61E-03	0.09	4.8E-01	7.48E-01	2.48	1.32E-02	3.17E-02
3-methoxytyrosine	0.35	3.28E-03	1.45E-02	0.21	8.80E-02	3.11E-01	1.2	2.32E-01	2.78E-01
methionine sulfoxide	-0.37	2.03E-03	9.80E-03	-0.27	3.35E-02	1.78E-01	-0.91	3.64E-01	3.97E-01
citrulline	-0.68	1.84E-10	4.87E-09	-0.52	1.12E-05	5.92E-04	-2.04	4.16E-02	8.66E-02

Amines are ranked based on the strength of their Pearson correlation coefficients (R) between CSF and plasma levels. For each amine, the correlation coefficient (R), associated *p*-values, and FDR-adjusted *q*-values are provided separately for TgF344-AD and WT rats. Positive R values indicate positive correlation between plasma and CSF concentrations of the amines. The Fisher’s z-value represent the statistical difference in correlation strength between TgF344-AD and WT rats. The amines are ranked ordered for the p-value in AD rats

### Age- and sex-dependent correlation patterns between CSF and plasma amines in TgF344-AD and WT rats

**Fig. 4:**
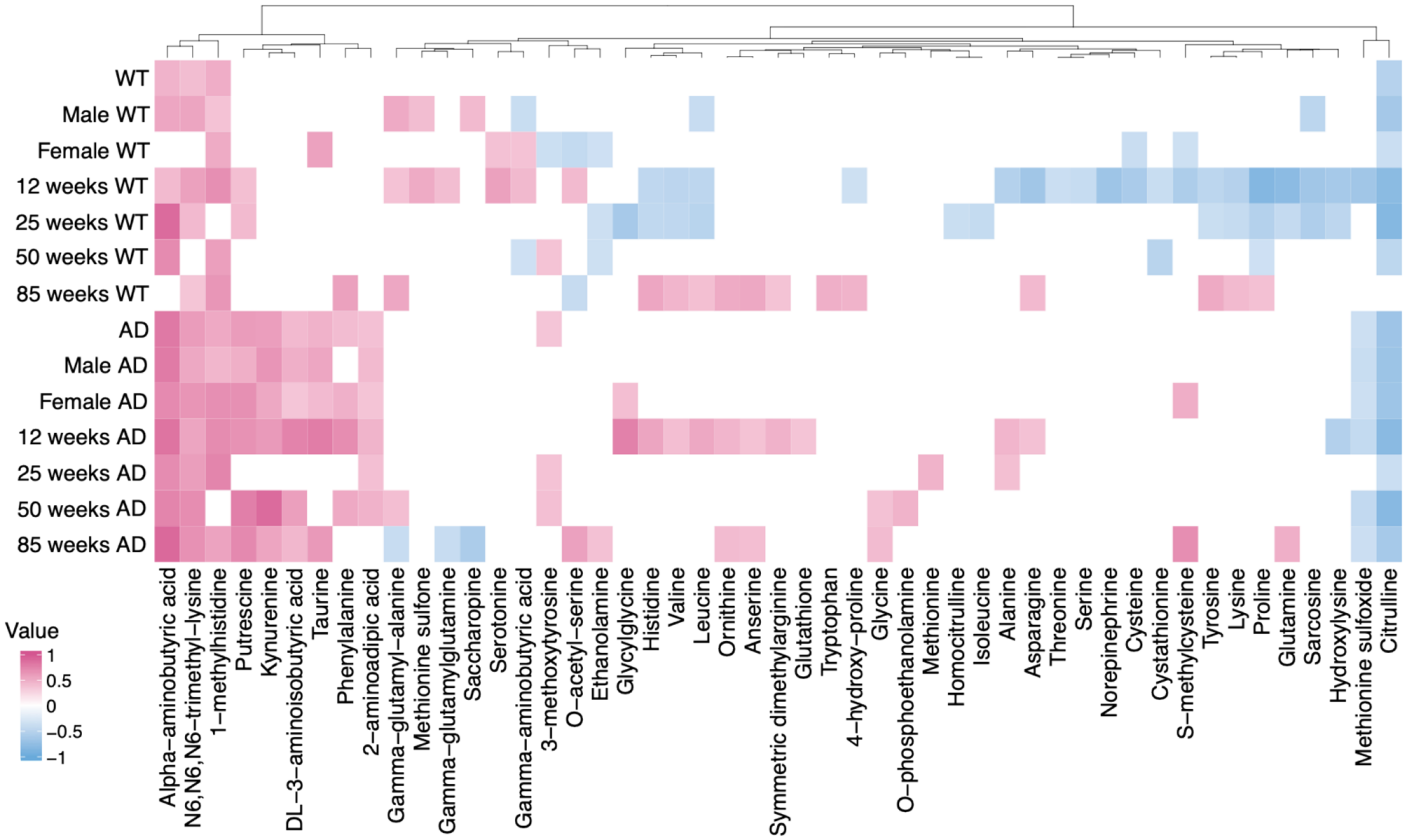
The heatmap illustrates the Pearson correlation results between metabolites in plasma and CSF across different age groups in TgF344-AD rats and WT rats. The plot highlights correlation patterns on the absolute correlation coefficient (|R|). Correlations with |R| ≥ 0.35 are displayed, with positive correlations shown in red and negative correlations in blue, while correlations with |R| < 0.35 were considered as too weak and blanked out. A full heatmap including all data is provided in Supplementary Fig. 1 for reference, and an alphabetically ordered version is shown in Supplementary Fig. 2

To visualize the correlations between plasma and CSF amines in TgF344-AD and WT rats across all four age groups (12, 25, 50, and 85 weeks), Pearson correlation coefficients were calculated and visualized as a clustered heatmap (Fig. 4). The heatmap reveals distinct clustering patterns of amines according to genotype, age, and sex, highlighting the progressive and sex-dependent alterations in plasma-CSF associations.

#### Age- and genotype-associated correlation patterns

At *12 weeks*, TgF344-AD rats showed strong positive plasma-CSF correlations for several amines, including 2-aminoadipic acid, DL-3-aminoisobutyric acid, kynurenine, phenylalanine, putrescine, and taurine compared with WT rats. At *25 weeks*, TgF344-AD rats continued to show relatively stronger positive correlations for 1-methylhistidine, alpha-aminobutyric acid, and N6,N6,N6-trimethyl-lysine. By *50 weeks*, positive correlations persisted for several amines such as alpha-aminobutyric acid, kynurenine, and putrescine in TgF344-AD rats. At *85 weeks*, WT rats exhibited stronger correlations for a different set of amines (e.g., 4-hydroxy-proline, asparagine, histidine, leucine, lysine, proline, symmetric dimethylarginine, tryptophan, tyrosine, and valine), while TgF344-AD rats showed weaker correlations.

#### Sex-specific plasma-CSF correlations

Descriptive sex-associated correlation patterns between TgF344-AD rats and WT rats were observed. In TgF344-AD rats, strong positive correlations (|R| ≥ 0.6) were found in females for metabolites such as glycylglycine, s-methylcysteine, and phenylalanine, which were absent in males. In WT rats, 3-methoxytyrosine, ethanolamine, o-acetyl-serine, serotonin, and taurine exhibited strong correlations in females but not in males, reflecting sex-linked metabolic coupling between plasma and CSF.

#### Cluster-based classification of correlation profiles

Based on the similarity of correlation profiles across genotype, age, and sex groups, three descriptive correlation patterns were observed. The first group showed significant correlations in both WT and TgF344-AD rats but differed in their strength. This group included 1-methylhistidine, alpha-aminobutyric acid, and N6,N6,N6-trimethyl-lysine. The second group mainly showed correlations in the TgF344-AD rats, but not in the WT rats, and consisted of 2-aminoadipic acid, DL-3-aminoisobutyric acid, kynurenine, putrescine, phenylalanine, and taurine. The third group only showed negative correlations in WT rats at 12 weeks of age, and consisted of alanine, asparagine, citrulline, cysteine, cystathionine, glutamine, hydroxylysine, lysine, methionine sulfoxide, norepinephrine, proline, sarcosine, serine, s-methylcysteine, threonine, and tyrosine.

## Discussion

Altering amine profiles in CSF and plasma are of particular interest in the early stages of AD progression. In this study, we investigated these changes using TgF344-AD and WT rats, assessing the influence of age and sex, as well as the relationships between the CSF and plasma amines. Male TgF344-AD rats showed earlier changes in amine levels at 25 weeks, whereas female rats showed changes at a later stage (50 weeks). Moreover, correlations between plasma and CSF amine profiles tended to be stronger in TgF344-AD rats compared to WT rats, suggesting differences in plasma-CSF associations under AD conditions. However, these correlations cannot be directly interpreted as changes in barrier permeability or transport function; their strength may instead reflect systemic co-regulation, shared metabolic pathways, or whole-body metabolic adaptations, rather than altered transport from peripheral regulation. Together, these findings describe age- and sex- associated patterns in amine metabolism in AD, highlighting the importance of explicitly addressing these factors, which is typically lacking in literature. The most prominent amine alterations are discussed in detail below.

### Early metabolic disruptions in male TgF344-AD rats involving oxidative stress, neuroinflammation, and neurotransmitter imbalance

The observed elevation of alpha-aminobutyric acid, asparagine, glycylproline, and methionine sulfone in the CSF of 25-week-old male TgF344-AD rats may suggest metabolic alterations associated with early-stage AD progression. Alpha-aminobutyric acid, linked to GABAergic systems [25], and asparagine, involved in nitrogen metabolism and neurotransmitter homeostasis [26, 27], may be related to changes in neurotransmitter balance at early disease stages. Glycylproline, a dipeptide derived from collagen degradation, has been associated with neuroinflammation and blood-brain barrier (BBB)-related processes in TgF344-AD rats [28]. Reduced GABA levels were observed at 50 weeks in female TgF344-AD rats in CSF, and plasma GABA levels were also reduced at the same time point when both sexes were analyzed together. GABA represents the principal inhibitory neurotransmitter in the central nervous system and, together with glutamate as the primary excitatory neurotransmitter, is critical for maintaining excitatory-inhibitory (E/I) balance [29–31]. Disruption of this balance has been widely implicated in AD pathology, contributing to synaptic dysfunction, neuronal hyperexcitability, and cognitive decline [32–34]. Although glutamate alterations were not among the most prominent changes observed in our dataset, the detected shifts in GABA and related metabolites may indicate alterations in neurotransmitter homeostasis during disease progression. Elevated glycylproline in male TgF344-AD rats may be consistent with increased neuroinflammatory responses, as AD is characterized by heightened neuroinflammation and microglial activation, particularly in male subjects [35]. Methionine sulfone, an oxidative product of methionine, accumulated in the CSF of male TgF344-AD rats, which may be indicative of increased oxidative burden at 25 weeks, an early disease stage [36, 37]. This observation is consistent with previous human metabolomics findings, where oxidative stress related pathways, including those involving methionine sulfone, were found altered in both plasma and CSF of AD patients [20, 36, 38–40]. Further, the five metabolites associated with the transsulfuration pathway (cystathionine, cysteine, serine, s-methylcysteine, threonine) showed strong negative correlations across matrices in 12-week-old WT rats but not in TgF344-AD rats. This difference may reflect early alterations in pathway regulation, although further validation is required. The transsulfuration pathway is critical for the maintenance of sulfur amino acid homeostasis with disruptions to this pathway being linked to neurodegenerative diseases, including AD [41, 42]. However, given the correlational nature of the present analysis, no mechanistic conclusions can be drawn, and further validation is required.

Our findings support methionine sulfone as a sensitive indicator of oxidative imbalance in male TgF344-AD rats and are in line with prior studies reporting stronger oxidative stress signatures in males than in females in TgF344-AD model, potentially reflecting sex differences in antioxidant defense mechanisms [43].

### Sex-specific reductions in polyamine-related pathways at mid-stage TgF344-AD in female rats

The observed decreases in DL-3-aminoisobutyric acid, gamma-aminobutyric acid, ornithine, and putrescine in the CSF of female TgF344-AD rats at 50 weeks indicate patterns that diff from those observed in males, suggesting sex-associated differences during later disease stage. Notably, DL-3-aminoisobutyric acid (also known as β-aminoisobutyric acid) has been genetically linked to aging-related mild cognitive impairment, further supporting its relevance to neurodegenerative processes [44]. Ornithine and putrescine, key metabolites in polyamine metabolism and cellular stress responses [45–48], may reflect changes in cellular stress responses or metabolic regulation [49, 50], although their precise role in AD progression remains to be determined. Changes in polyamine metabolism have previously been associated with neuronal survival, regulation of oxidative stress, and modulation of neuroinflammation [45, 47], highlighting the potential relevance of these amines in maintaining brain function during neurodegeneration.

### Differences in plasma-CSF amine correlations in TgF344-AD rats

We observed plasma-CSF correlations with |R| ≥ 0.4 for specific amines, including 1-methylhistidine, alpha-aminobutyric acid, citrulline, DL-3-aminoisobutyric acid, N6,N6,N6-trimethyl-lysine, phenylalanine, putrescine taurine, and kynurenine. Among these, 1-methylhistidine showed relatively consistent correlations between plasma and CSF in both WT and TgF344-AD rats across age and sex, whereas the other amines tended to show stronger or more apparent correlations mainly in TgF344-AD rats. In particular, alpha-aminobutyric acid, N6,N6,N6-trimethyl-lysine, and putrescine showed relatively strong correlations in TgF344-AD rats (|R| ≥ 0.6), while the corresponding correlations in WT rats were generally in the moderate range. Citrulline also showed a relatively strong negative correlation in TgF344-AD rats compared with a more moderate negative correlation in WT rats.

The stronger plasma-CSF correlations in TgF344-AD rats may reflect differences in the relationships between systemic and central metabolic processes. However, correlation analyses alone cannot distinguish altered transport from systemic co-regulation or whole-body metabolic adaptations. AD pathology is characterized by neuroinflammation and oxidative stress, processes known to influence both central and peripheral metabolism, which may be relevant to the observed correlations patterns [51–56].

Several of the amines showing stronger plasma-CSF correlations in TgF344-AD rats are known to be exchanged between peripheral and central compartments under physiological conditions with intact BBB function. For instance, 2-aminoadipic acid is transported across the BBB via system y + and system L transporters [12, 57]. Kynurenine crosses the BBB via the L-type amino acid transporter 1 (LAT1), and has been implicated in neuroinflammation processes [58–61]. Taurine has been described as a neuroprotective and anti-inflammatory amino acid [62–64]. These examples illustrate known physiological transport routes; however, the present data do not provide evidence for altered transporter activity or barrier function. Therefore, any mechanistic interpretation related to transport should be considered speculative and require direct functional validation [65].

Furthermore, Fisher’s r-to-z transformation analyses revealed differences in the strength of plasma-CSF correlations between TgF344-AD and WT groups (Supplementary Table 5). Alpha-aminobutyric acid showed a stronger positive correlation in TgF344-AD rats than in WT rats (*R* = 0.80 vs. 0.46), and N6,N6,N6-trimethyl-lysine also showed a stronger positive correlation in TgF344-AD rats (*R* = 0.60 vs. 0.40). Putrescine and kynurenine followed a similar pattern, with relatively strong positive correlations in TgF344-AD rats (both *R* ≈ 0.60) but much weaker correlations in WT rats (both *R* ≈ 0.20). In contrast, citrulline showed a stronger negative correlation in TgF344-AD rats than in WT rats (*R* = -0.68 vs. -0.52). Sex- and age-associated patterns were also observed; however, these findings should be interpreted as descriptive observations rather than statistically confirmed interaction effects. Female rats showed larger AD-WT differences for several amines, particularly alpha-aminobutyric acid (R_AD_ = 0.79, R_WT_ = 0.54 in male rats, R_AD_ = 0.7, R_WT_ = 0.09 in female rats), citrulline (R_AD_ = -0.69, R_WT_ = -0.63 in male rats, R_AD_ = -0.68, R_WT_ = -0.38 in female rats), and N6,N6,N6-trimethyl-lysine (R_AD_=0.53, R_WT_ =0.54 in male rats, R_AD_=0.64, R_WT_ =0.25 in female rats) (Supplementary Tables 6–7), whereas citrulline displayed dynamic age-related variation, with stronger negative correlations in WT rats at 25 weeks (R_WT_ =-0.84) and in TgF344-AD rats at 50 (R_AD_=-0.82) and 85 weeks (R_AD_=-0.63) (Supplementary Tables 8–11).

The observed differences in plasma-CSF amine associations highlight the importance of considering sex as a biological variable, although no definitive sex-dependent effects can be concluded from the present data. Future studies are needed to further investigate the biological relevance of these observations. While the observed plasma-CSF associations suggest potential utility for biomarker exploration, their interpretation should remain cautious given the correlational nature of the analysis.

Importantly, AD is increasingly recognized as a systemic disorder involving peripheral inflammation and metabolic dysregulation [66–68]. Therefore, alterations in plasma amine concentrations may reflect systemic metabolic adaptations rather than exclusively brain-specific processes. Strong plasma-CSF correlations could thus arise from coordinated systemic regulation affecting both compartments, rather than direct changes in barrier transport [69]. Notably, peripheral organs from the same animals have also been collected and will be subjected to future multi-omics analyses, which may help to better understand the systemic metabolic alterations underlying these observations.

### Advantages of the TgF344-AD model in translational studies

This TgF344-AD rat model offers unique opportunities for experimental interventions, including: (i) Tissue-specific pathophysiology studies enabling a deeper investigation into brain-periphery metabolic interactions. (ii) Therapeutic testing allowing for targeted modulation of metabolic pathways through pharmacological interventions. (iii) Mechanistic validation providing opportunities to apply inhibitors or genetic modifications to dissect key disease-related pathways. Thus, our study describes amine alterations associated with AD and highlights the potential of the TgF344-AD rat model as a translational tool, while further studies are required to establish mechanistic relevance and therapeutic applicability.

### Limitations of the study

This study exclusively analyzed amines, thereby capturing only a subset of the broader metabolic alterations associated with AD progression and aging. Other important metabolite classes, such as lipids, neurotransmitters, and proteins, were not assessed. Additionally, the experimental design included four discrete age time points (12, 25, 50, and 85 weeks), which allowed investigation of broad age-related trends but may have overlooked intermediate metabolic changes. A more frequent sampling strategy could provide finer temporal resolution and additional insights into dynamic metabolic transitions. Importantly, this study did not include direct measurements of BBB or blood-CSF barrier integrity. Therefore, plasma-CSF correlations should not be interpreted as direct evidence of altered barrier permeability or transporter function. Moreover, correlation analyses cannot distinguish transport-related changes from systemic co-regulation or whole-body metabolic adaptations.

### Implications and future directions

Our findings have implications for both mechanistic and translational AD research. The observed amine level changes across age, sex and disease status, together with their altered plasma-CSF correlation patterns, suggest potential dysregulation in systemic amine metabolism, which may be related to processes such as neurotransmission, inflammation, and oxidative stress. However, these interpretations remain associative and require further validation.

Given the parallels between our findings in TgF344-AD rats and prior human studies, this model may serve as a valuable platform for investigating AD-related processes. For example, Basun et al. [16] reported significantly decreased plasma levels of glutamate and taurine, and increased CSF levels of glycine, leucine, and valine in AD patients compared to healthy controls. These human findings closely mirror our results in TgF344-AD rats, supporting the translational relevance of the model.

Additionally, several amines showed correlations between CSF and plasma, indicating potential relevance for biomarkers exploration. However, these associations should be interpreted cautiously, as the present study does not provide direct evidence for transporter activities or blood- CSF/brain barrier function. Future research should focus on validating these findings in human cohorts and further investigating the biological processes underlying amine regulation.

Finally, the observed differences between sexes highlight the importance of considering sex as a biological variable in AD research. However, no definitive sex-dependent effects can be concluded based on the present data, and further studies are needed to clarify the biological basis of these observations.

## Conclusions

Overall, our findings provide insights into age-, sex-, and disease-associated differences in amine levels in plasma and CSF in the TgF344-AD rat model. These results describe patterns of amine dysregulation in AD and highlight the importance of considering both biological sex and disease stage in future studies. The TgF344-AD rat model represents a valuable tool for translational research, although further validation is required to establish mechanistic links and clinical applicability.

## Supplementary Information

Below is the link to the electronic supplementary material.

Supplementary Material 1

## Data Availability

All data analyzed in this study are available upon reasonable request.

## Abbreviations

AD: Alzheimer’s disease
CSF: Cerebrospinal fluid
WT: Wildtype
GABA: Gamma-aminobutyric acid
BCAAs: Branched-chain amino acids
EDTA: Ethylenediaminetetraacetic acid
BMFL: Biomedical Metabolomics Facility Leiden
MRM: Multiple Reaction Monitoring
FDR: False discovery rate
GLM: Generalized linear regression models
BBB: Blood-brain barrier
LAT: L-type amino acid transporter
