# Supplementary material for: Longitudinal metabolomic profiling of biogenic amines in plasma and CSF, and their correlation, reveals sex-specific and age changes in TgF344 Alzheimer’s disease transgenic and wildtype rats
Source: Fluids Barriers CNS. 2026 May 9;23:69. doi: 10.1186/s12987-026-00811-8 (PMC13162435; doi:10.1186/s12987-026-00811-8)
Supplement: Supplementary file 1 — Supplementary Material 1 [file 12987_2026_811_MOESM1_ESM.zip › Supplementary materials/Supplementary Tables 5-11.docx]

To provide a more detailed overview of the metabolite-specific plasma-CSF correlation patterns, supplementary Tables 3-9 summarize the pearson correlation coefficients for each metabolite across TgF344-AD, WT, and combined (ADWT) groups, grouped by sex (Supplementary Tables 4-5) and age (Supplementary Tables 6-9). For each group, the Fisher’s z-transformed correlation coefficients, corresponding p-values, and FDR-adjusted q-values are provided. Only metabolites that showed a statistically supported correlation (|R| > 0.35, p < 0.05) in at least one group (e.g., TgF344-AD, WT, or ADWT, stratified by age or sex) were included in the supplementary table. Results are rounded to two decimal places, and non-significant comparisons (p > 0.05) are labeled as “n.s.”

**Supplementary Table 5.** The correlation coefficient between CSF and plasma of the metabolites within AD, WT or a combination of AD and WT (ADWT) and the fisher's transformed z-score of the AD correlation coefficient (n = 136) and WT correlation coefficient (n = 128) for **all** rats. Only the metabolites that have at least one significant (^*^p<0.05) correlation coefficient higher than |0.35| in AD, WT and/or ADWT are displayed in the table. It is rounded in two digits. The p-value and FDR adjusted p-value (q-value) are calculated with a t-test. (^ns^p>0.05)

|  | Correlation coefficient | | | Fisher's Z-value AD/WT | | |
| --- | --- | --- | --- | --- | --- | --- |
| Amine | R_AD_ | R_WT_ | R_(ADWT)_ | z-value | p-value | q-value |
| 1-methylhistidine | 0.52 | 0.49 | 0.51 | 0.23 | 8.17E-01 | 8.17E-01 |
| 2-aminoadipic acid | 0.38 | 0.09^ns^ | 0.24 | 1.74 | 8.18E-02 | 1.40E-01 |
| 3-methoxytyrosine | 0.35 | 0.21^ns^ | 0.28 | 0.85 | 3.95E-01 | 5.27E-01 |
| alpha-aminobutyric acid | 0.80 | 0.46 | 0.63 | 3.37 | 7.43E-04 | 2.97E-03 |
| Citrulline | -0.68 | -0.52 | -0.60 | -1.42 | 1.56E-01 | 2.34E-01 |
| DL-3-aminoisobutyric acid | 0.42 | -0.19^ns^ | 0.17^ns^ | 3.59 | 3.33E-04 | 2.00E-03 |
| Kynurenine | 0.59 | 0.20^ns^ | 0.45 | 2.66 | 7.73E-03 | 1.86E-02 |
| Methionine sulfoxide | -0.37 | -0.27 | -0.32 | -0.63 | 5.28E-01 | 6.33E-01 |
| N6,N6,N6-trimethyl lysine | 0.60 | 0.40 | 0.52 | 1.51 | 1.31E-01 | 2.25E-01 |
| Phenylalanine | 0.41 | 0.07^ns^ | 0.26 | 2.06 | 3.94E-02 | 7.88E-02 |
| Putrescine | 0.60 | 0.20^ns^ | 0.42 | 2.75 | 5.92E-03 | 1.78E-02 |
| Taurine | 0.47 | 0.28 | 0.39 | 1.25 | 2.11E-01 | 3.17E-01 |

**Supplementary Table 6.** The correlation coefficient between CSF and plasma of the metabolites within AD, WT or a combination of AD and WT (ADWT) and the fisher's transformed z-score of the AD correlation coefficient (n = 62) and WT correlation coefficient (n = 72) for the **male** rats. Only the metabolites that have at least one significant (^*^p<0.05) correlation coefficient higher than |0.35| in AD, WT and/or ADWT are displayed in the table. It is rounded in two digits. The p-value and FDR adjusted p-value (q-value) are calculated with a t-test. (^ns^p>0.05)

|  | Correlation coefficient | | | Fisher's Z-value AD/WT | | |
| --- | --- | --- | --- | --- | --- | --- |
| Amine | R_AD_ | R_WT_ | R_(ADWT)_ | z-value | p-value | q-value |
| 1-methylhistidine | 0.44 | 0.37 | 0.42 | 0.36 | 7.17E-01 | 8.60E-01 |
| 2-aminoadipic acid | 0.42 | -0.03^ns^ | 0.22^ns^ | 1.85 | 6.41E-02 | 1.47E-01 |
| 3-methoxytyrosine | 0.28^ns^ | 0.33 | 0.31 | -0.24 | 8.14E-01 | 8.61E-01 |
| Alpha-aminobutyric acid | 0.79 | 0.54 | 0.67 | 1.87 | 6.09E-02 | 1.47E-01 |
| Citrulline | -0.69 | -0.63 | -0.65 | -0.39 | 6.95E-01 | 8.60E-01 |
| DL-3-aminoisobutyric acid | 0.49 | -0.32^ns^ | 0.14^ns^ | 3.34 | 8.43E-04 | 1.52E-02 |
| Gamma-aminobutyric acid | 0.06^ns^ | -0.39 | -0.18^ns^ | 1.83 | 6.72E-02 | 1.47E-01 |
| Gamma glutamyl alanine | 0.03^ns^ | 0.51 | 0.26 | -2.09 | 3.64E-02 | 1.47E-01 |
| Gamma-glutamyl glutamine | -0.04^ns^ | 0.34 | 0.15^ns^ | -1.52 | 1.29E-01 | 1.93E-01 |
| Kynurenine | 0.64 | 0.31^ns^ | 0.53 | 1.7 | 8.95E-02 | 1.47E-01 |
| Leucine | 0.04^ns^ | -0.4 | -0.13^ns^ | 1.81 | 7.11E-02 | 1.47E-01 |
| Methionine sulfone | -0.02^ns^ | 0.4 | 0.16^ns^ | -1.74 | 8.27E-02 | 1.47E-01 |
| Methionine.sulfoxide | -0.39 | -0.34 | -0.37 | -0.24 | 8.11E-01 | 8.61E-01 |
| N6,N6,N6-trimethyl lysine | 0.53 | 0.54 | 0.54 | -0.08 | 9.40E-01 | 9.40E-01 |
| Putrescine | 0.49 | 0.22^ns^ | 0.35 | 1.2 | 2.29E-01 | 3.17E-01 |
| Saccharopine | -0.18^ns^ | 0.42 | 0.08^ns^ | -2.47 | 1.37E-02 | 1.23E-01 |
| Sarcosine | -0.06^ns^ | -0.48 | -0.24 | 1.77 | 7.60E-02 | 1.47E-01 |
| Taurine | 0.54 | 0.17^ns^ | 0.36 | 1.7 | 9.01E-02 | 1.47E-01 |

**Supplementary Table 7.** The correlation coefficient between CSF and plasma of the metabolites within AD, WT or a combination of AD and WT (ADWT) and the fisher's transformed z-score of the AD correlation coefficient (n = 74) and WT correlation coefficient (n = 56) for the **female** rats. Only the metabolites that have at least one significant (^*^p<0.05) correlation coefficient higher than |0.35| in AD, WT and/or ADWT are displayed in the table. It is rounded in two digits. The p-value and FDR adjusted p-value (q-value) are calculated with a t-test. (^ns^p>0.05)

|  | Correlation coefficient | | | Fisher's Z-value AD/WT | | |
| --- | --- | --- | --- | --- | --- | --- |
| Amine | R_AD_ | R_WT_ | R_(ADWT)_ | z-value | p-value | q-value |
| 1-methylhistidine | 0.68 | 0.51 | 0.55 | 1.01 | 3.12E-01 | 5.04E-01 |
| 2-aminoadipic acid | 0.36 | 0.31^ns^ | 0.29 | 0.21 | 8.31E-01 | 8.72E-01 |
| 3-methoxytyrosine | -0.14^ns^ | -0.37^ns^ | -0.27 | 0.94 | 3.48E-01 | 5.21E-01 |
| Alpha-aminobutyric acid | 0.70 | 0.09^ns^ | 0.37 | 2.95 | 3.18E-03 | 3.34E-02 |
| Citrulline | -0.68 | -0.38 | -0.53 | -1.63 | 1.03E-01 | 2.17E-01 |
| Cysteine | 0.04^ns^ | -0.38 | -0.21^ns^ | 1.67 | 9.48E-02 | 2.17E-01 |
| DL-3-aminoisobutyric acid | 0.35 | 0.05^ns^ | 0.23^ns^ | 1.2 | 2.31E-01 | 4.19E-01 |
| Gamma-aminobutyric acid | 0.34 | 0.37 ^ns^ | 0.34 | -0.13 | 8.96E-01 | 8.96E-01 |
| Glycylglycine | 0.39 | -0.02^ns^ | 0.23^ns^ | 1.64 | 1.01E-01 | 2.17E-01 |
| Kynurenine | 0.53 | 0.12^ns^ | 0.40 | 1.78 | 7.47E-02 | 2.17E-01 |
| Methionine sulfone | 0.29^ns^ | 0.18 ^ns^ | 0.25 | 0.44 | 6.58E-01 | 7.68E-01 |
| Methionine sulfoxide | -0.36 | -0.18^ns^ | -0.28 | -0.74 | 4.59E-01 | 6.33E-01 |
| N6,N6,N6-trimethyl-lysine | 0.64 | 0.25^ns^ | 0.50 | 1.91 | 5.64E-02 | 2.17E-01 |
| O-acetyl-serine | 0.08^ns^ | -0.42 | -0.17^ns^ | 2 | 4.51E-02 | 2.17E-01 |
| O-phosphoethanolamine | 0.32^ns^ | 0.25^ns^ | 0.31 | 0.29 | 7.72E-01 | 8.54E-01 |
| Phenylalanine | 0.48 | 0.09^ns^ | 0.31 | 1.68 | 9.28E-02 | 2.17E-01 |
| Putrescine | 0.67 | 0.18^ns^ | 0.46 | 2.39 | 1.70E-02 | 1.19E-01 |
| S-methylcysteine | 0.49 | -0.35^ns^ | 0.06^ns^ | 3.42 | 6.22E-04 | 1.31E-02 |
| Serotonine | 0.09^ns^ | 0.38 | 0.22^ns^ | -1.18 | 2.40E-01 | 4.19E-01 |
| Symmetric dimethylarginine | 0.34 | 0.23^ns^ | 0.27 | 0.46 | 6.49E-01 | 7.68E-01 |
| Taurine | 0.42 | 0.56 | 0.47 | -0.7 | 4.82E-01 | 6.33E-01 |

**Supplementary Table 8.** The correlation coefficient between CSF and plasma of the metabolites within AD, WT or a combination of AD and WT (ADWT) and the fisher's transformed z-score of the AD correlation coefficient (n= 26) and WT correlation coefficient (n = 24) for the **12** weeks old rats. Only the metabolites that have at least one significant (^*^p<0.05) correlation coefficient higher than |0.35| in AD, WT and/or ADWT are displayed in the table. It is rounded in two digits. The p-value and FDR adjusted p-value (q-value) are calculated with a t-test. (^ns^p>0.05)

|  | Correlation coefficient | | | Fisher's Z-value AD/WT | | |
| --- | --- | --- | --- | --- | --- | --- |
| Amine | R_AD_ | R_WT_ | R_(ADWT)_ | z-value | p-value | q-value |
| 1-methylhistidine | 0.70 | 0.67 | 0.67 | 0.11 | 9.15E-01 | 9.30E-01 |
| Alpha-aminobutyric acid | 0.84 | 0.42^ns^ | 0.64 | 1.92 | 5.51E-02 | 1.75E-01 |
| Asparagine | 0.38^ns^ | -0.66 | -0.08^ns^ | 2.12 | 3.41E-02 | 1.04E-01 |
| Citrulline | -0.81 | -0.80 | -0.76 | -0.09 | 9.30E-01 | 9.30E-01 |
| Cysteine | 0.32^ns^ | -0.60 | -0.22^ns^ | 2.07 | 3.87E-02 | 1.04E-01 |
| DL-3-aminoisobutyric acid | 0.74 | -0.21^ns^ | 0.21^ns^ | 2.2 | 2.79E-02 | 8.83E-02 |
| Glutamine | -0.15^ns^ | -0.80 | -0.34^ns^ | 2.17 | 3.02E-02 | 8.83E-02 |
| Glycylglycine | 0.76 | -0.34^ns^ | 0.27^ns^ | 2.42 | 1.56E-02 | 7.41E-02 |
| Hydroxylysine | -0.54^ns^ | -0.63 | -0.58 | 0.33 | 7.40E-01 | 8.79E-01 |
| Kynurenine | 0.61 | 0.3^ns^ | 0.51 | 0.99 | 3.22E-01 | 5.10E-01 |
| Methionine sulfoxide | -0.43^ns^ | -0.66 | -0.52 | 0.78 | 4.36E-01 | 6.20E-01 |
| N6,N6,N6-trimethyl lysine | 0.55^ns^ | 0.57^ns^ | 0.56 | -0.11 | 9.11E-01 | 9.30E-01 |
| Norepinephrine | -0.32^ns^ | -0.68 | -0.36^ns^ | 1.44 | 1.50E-01 | 2.96E-01 |
| Phenylalanine | 0.69 | -0.31^ns^ | 0.29^ns^ | 2.09 | 3.66E-02 | 1.04E-01 |
| Proline | 0.15^ns^ | -0.84 | -0.42 | 2.62 | 8.75E-03 | 7.41E-02 |
| Putrescine | 0.66 | 0.39^ns^ | 0.52 | 1.08 | 2.80E-01 | 4.69E-01 |
| Sarcosine | -0.13^ns^ | -0.66 | -0.44 | 1.46 | 1.45E-01 | 2.96E-01 |
| S-methylcysteine | 0.11^ns^ | -0.58 | -0.15^ns^ | 1.24 | 0.216 | 0.407 |
| Taurine | 0.78 | 0.26^ns^ | 0.57 | 1.62 | 1.05E-01 | 2.51E-01 |

**Supplementary Table 9.** The correlation coefficient between CSF and plasma of the metabolites within AD, WT or a combination of AD and WT (ADWT) and the fisher's transformed z-score of the correlation coefficient of AD (n = 38) and the correlation coefficient of WT (n = 36) for the **25** weeks old rats. Only the metabolites that have at least one significant (^*^p<0.05) correlation coefficient higher than |0.35| in AD, WT and/or ADWT are displayed in the table. It is rounded in two digits. The p-value and FDR adjusted p-value (q-value) are calculated with a t-test. (^ns^p>0.05)

|  | Correlation coefficient | | | Fisher's Z-value AD/WT | | |
| --- | --- | --- | --- | --- | --- | --- |
| Amine | R_AD_ | R_WT_ | R_(ADWT)_ | z-value | p-value | q-value |
| 1-methylhistidine | 0.74 | 0.11^ns^ | 0.45 | 2.34 | 1.94E-02 | 8.19E-02 |
| 3-methoxytyrosine | 0.37^ns^ | 0.34^ns^ | 0.34 | 0.1 | 9.24E-01 | 9.24E-01 |
| Alpha-aminobutyric acid | 0.69 | 0.88 | 0.82 | -1.47 | 1.42E-01 | 2.23E-01 |
| Citrulline | -0.38^ns^ | -0.84 | -0.64 | 2.28 | 2.23E-02 | 8.19E-02 |
| Glycylglycine | 0.14^ns^ | -0.62 | -0.10^ns^ | 2.41 | 1.60E-02 | 8.19E-02 |
| Hydroxylysine | -0.18^ns^ | -0.47 | -0.25^ns^ | 0.91 | 3.61E-01 | 4.97E-01 |
| Leucine | 0.03^ns^ | -0.50 | -0.25^ns^ | 1.61 | 1.07E-01 | 2.23E-01 |
| Methionine | 0.46 | 0.23^ns^ | 0.18^ns^ | 0.73 | 4.64E-01 | 5.67E-01 |
| N6,N6,N6-trimethyl lysine | 0.59 | 0.42^ns^ | 0.49 | 0.64 | 5.22E-01 | 5.74E-01 |
| Proline | -0.06^ns^ | -0.53 | -0.39 | 1.47 | 1.40E-01 | 2.23E-01 |
| Sarcosine | -0.10^ns^ | -0.56 | -0.32^ns^ | 1.51 | 1.31E-01 | 2.23E-01 |

**Supplementary Table 10.** The correlation coefficient between CSF and plasma of the metabolites within AD, WT or a combination of AD and WT (ADWT) and the fisher's transformed z-score of the correlation coefficient in AD (n = 36) and the correlation coefficient in WT (n = 32) for the **50** weeks old rats. Only the metabolites that have at least one significant (^*^p<0.05) correlation coefficient higher than |0.35| in AD, WT and/or ADWT are displayed in the table. It is rounded in two digits. The p-value and FDR adjusted p-value (q-value) are calculated with a t-test. (^ns^p>0.05)

|  | Correlation coefficient | | | Fisher's Z-value AD/WT | | |
| --- | --- | --- | --- | --- | --- | --- |
| Amine | R_AD_ | R_WT_ | R_(ADWT)_ | z-value | p-value | q-value |
| 1-methylhistidine | 0.17^ns^ | 0.58 | 0.31^ns^ | -1.3 | 1.95E-01 | 3.07E-01 |
| 3-methoxytyrosine | 0.39^ns^ | 0.36^ns^ | 0.41 | 0.09 | 9.27E-01 | 9.27E-01 |
| Alpha-aminobutyric acid | 0.73 | 0.70 | 0.71 | 0.16 | 8.71E-01 | 9.27E-01 |
| Citrulline | -0.82 | -0.47^ns^ | -0.66 | -1.71 | 8.79E-02 | 1.93E-01 |
| DL-3-aminoisobutyric acid | 0.58 | -0.06^ns^ | 0.32^ns^ | 1.91 | 5.66E-02 | 1.71E-01 |
| Kynurenine | 0.88 | 0.18^ns^ | 0.61 | 3.15 | 1.63E-03 | 1.79E-02 |
| Methionine sulfone | 0.24^ns^ | 0.32^ns^ | 0.35 | -0.23 | 8.19E-01 | 9.27E-01 |
| Methionine sulfoxide | -0.44^ns^ | -0.27^ns^ | -0.37 | -0.52 | 6.06E-01 | 8.33E-01 |
| N6,N6,N6-trimethyl lysine | 0.69 | 0.34^ns^ | 0.53 | 1.3 | 1.92E-01 | 3.07E-01 |
| Phenylalanine | 0.52 | -0.13^ns^ | 0.25^ns^ | 1.87 | 6.20E-02 | 1.71E-01 |
| Putrescine | 0.77 | 0.02^ns^ | 0.44 | 2.64 | 8.29E-03 | 4.56E-02 |

**Supplementary Table 11.** The correlation coefficient between CSF and plasma of the metabolites within AD, WT or a combination of AD and WT (ADWT) and the fisher's transformed z-score of the correlation coefficient in AD (n = 36) and the correlation coefficient in WT (n = 36) for the **85** weeks old rats. Only the metabolites that have at least one significant (^*^p<0.05) correlation coefficient higher than |0.35| in AD, WT and/or ADWT are displayed in the table. It is rounded in two digits. The p-value and FDR adjusted p-value (q-value) are calculated with a t-test. (^ns^p>0.05)

|  | Correlation coefficient | | | Fisher's Z-value AD/WT | | |
| --- | --- | --- | --- | --- | --- | --- |
| Amine | R_AD_ | R_WT_ | R_(ADWT)_ | z-value | p-value | q-value |
| 1-methylhistidine | 0.56 | 0.63 | 0.60 | -0.3 | 7.66E-01 | 7.66E-01 |
| Alpha-aminobutyric acid | 0.89 | 0.13^ns^ | 0.43 | 3.54 | 4.06E-04 | 8.53E-03 |
| Anserine | 0.40^ns^ | 0.53 | 0.45 | -0.46 | 6.48E-01 | 7.66E-01 |
| Citrulline | -0.63 | 0.08^ns^ | -0.27^ns^ | -2.25 | 2.44E-02 | 1.03E-01 |
| Gamma glutamyl alanine | -0.40^ns^ | 0.53 | 0.16^ns^ | -2.78 | 5.50E-03 | 3.73E-02 |
| Glutamine | 0.48 | -0.19^ns^ | 0.23^ns^ | 1.96 | 5.01E-02 | 1.46E-01 |
| Histidine | 0.20^ns^ | 0.54 | 0.40 | -1.1 | 2.72E-01 | 4.39E-01 |
| Kynurenine | 0.54 | 0.1^ns^ | 0.33 | 1.38 | 1.68E-01 | 3.56E-01 |
| Leucine | 0.29^ns^ | 0.39^ns^ | 0.35 | -0.31 | 7.56E-01 | 7.66E-01 |
| Lysine | 0.29^ns^ | 0.42^ns^ | 0.37 | -0.41 | 6.83E-01 | 7.66E-01 |
| N6,N6,N6-trimethyl lysine | 0.67 | 0.35 ^ns^ | 0.53 | 1.22 | 2.23E-01 | 3.90E-01 |
| O-acetyl-serine | 0.57 | -0.41^ns^ | 0.04^ns^ | 2.97 | 3.01E-03 | 3.16E-02 |
| Ornithine | 0.41^ns^ | 0.51 | 0.48 | -0.35 | 7.28E-01 | 7.66E-01 |
| Phenylalanine | 0.31^ns^ | 0.57 | 0.43 | -0.9 | 3.71E-01 | 5.56E-01 |
| Putrescine | 0.71 | 0.13 ^ns^ | 0.41 | 2.07 | 3.83E-02 | 1.34E-01 |
| S-methylcysteine | 0.68 | 0.13^ns^ | 0.42 | 1.91 | 5.58E-02 | 1.46E-01 |
| Saccharopine | -0.58 | 0.31^ns^ | -0.24^ns^ | -2.69 | 7.10E-03 | 3.73E-02 |
| Taurine | 0.62 | 0.22^ns^ | 0.43 | 1.37 | 1.70E-01 | 3.56E-01 |
| Tryptophan | 0.31^ns^ | 0.48 | 0.40 | -0.55 | 5.79E-01 | 7.60E-01 |
| Tyrosine | 0.11^ns^ | 0.52 | 0.27^ns^ | -1.28 | 2.02E-01 | 3.86E-01 |
| Valine | 0.22^ns^ | 0.45^ns^ | 0.36 | -0.71 | 4.75E-01 | 6.65E-01 |
