# Supplementary figures and images for: Longitudinal metabolomic profiling of biogenic amines in plasma and CSF, and their correlation, reveals sex-specific and age changes in TgF344 Alzheimer’s disease transgenic and wildtype rats
Source: Fluids Barriers CNS. 2026 May 9;23:69. doi: 10.1186/s12987-026-00811-8 (PMC13162435; doi:10.1186/s12987-026-00811-8)

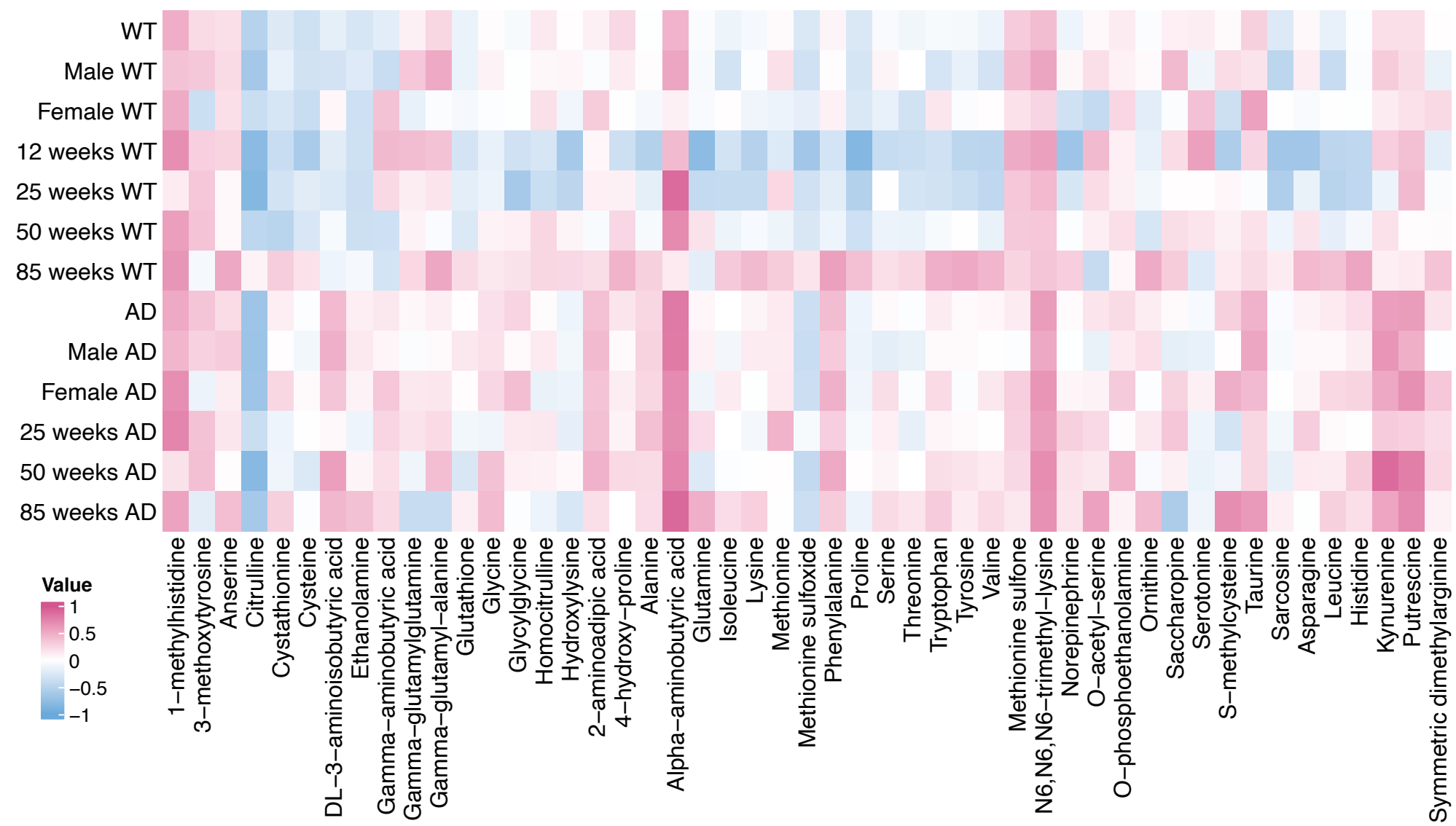

Supplement: Supplementary file 1 — Supplementary Material 1 [file 12987_2026_811_MOESM1_ESM.zip › Supplementary materials/Supplementary Figure 2.pdf]

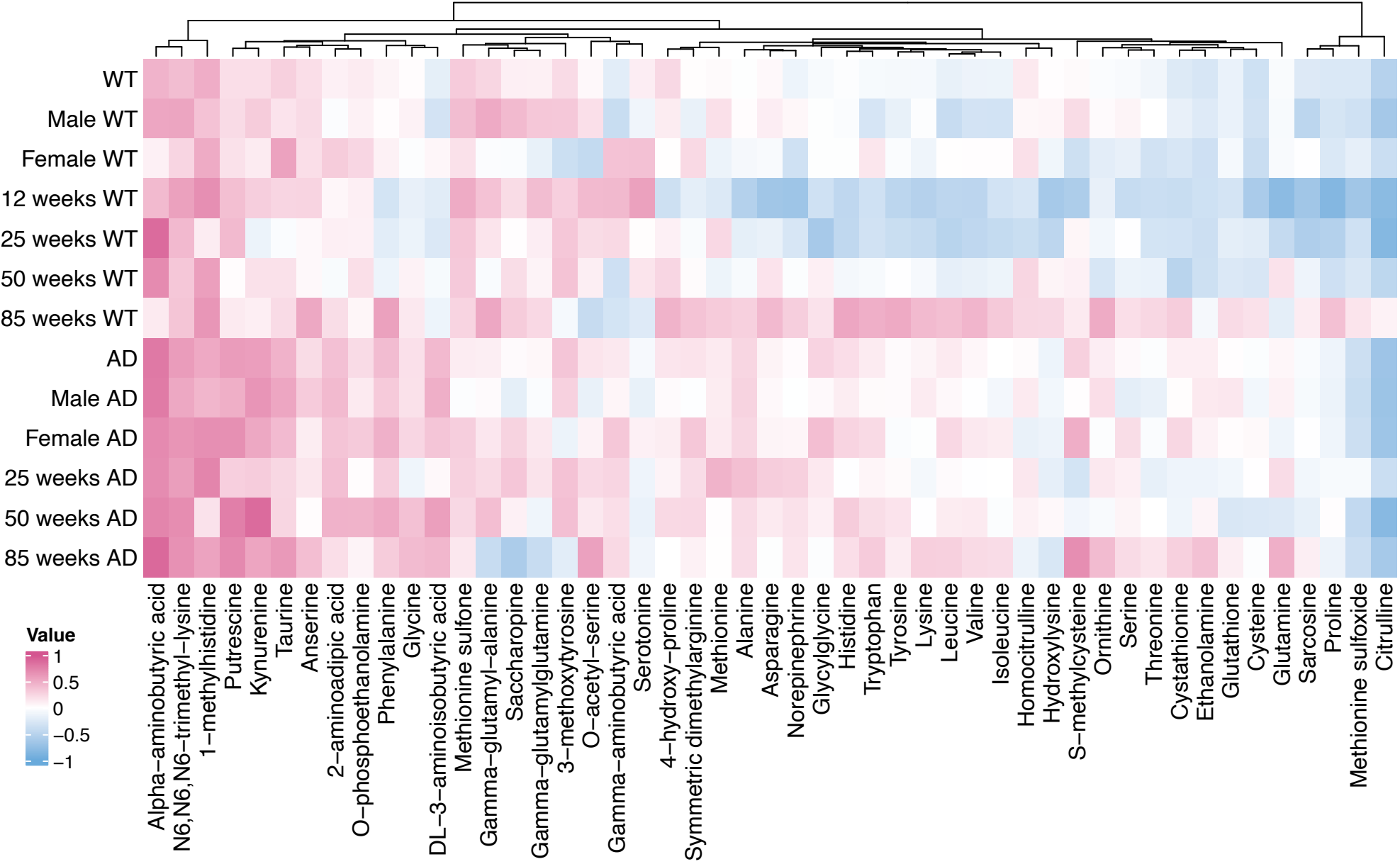

Supplement: Supplementary file 1 — Supplementary Material 1 [file 12987_2026_811_MOESM1_ESM.zip › Supplementary materials/Supplementary Figure 1.pdf]
